# Functional Regulatory Role of STAT3 in HPV16-Mediated Cervical Carcinogenesis

**DOI:** 10.1371/journal.pone.0067849

**Published:** 2013-07-18

**Authors:** Shirish Shukla, Sutapa Mahata, Gauri Shishodia, Arvind Pandey, Abhishek Tyagi, Kanchan Vishnoi, Seemi F. Basir, Bhudev C. Das, Alok C. Bharti

**Affiliations:** 1 Division of Molecular Oncology, Institute of Cytology and Preventive Oncology, NOIDA, Uttar Pradesh, India; 2 Department of Biosciences, Jamia Millia Islamia, New Delhi, India; 3 Dr. B.R. Ambedkar Centre for Biomedical Research, University of Delhi, New Delhi, India; Van Andel Institute, United States of America

## Abstract

Signal transducer and activator of transcription 3 (STAT3) is an oncogenic transcription factor constitutively active and aberrantly expressed in cervical cancer. However, the functional role of STAT3 in regulation of HPV's viral oncogene expression and downstream events associated with cervical carcinogenesis is not known. Our present study performed on HPV16-positive cervical cancer cell lines (SiHa and CaSki) and primary tumor tissues revealed a strong positive correlation of constitutively active STAT3 with expression of HPV16 E6 and E7 oncoproteins and a negative association with levels of p53 and pRB. Pharmacologic targeting of STAT3 expression in cervical cancer cell lines either by STAT3-specific siRNA or blocking its tyrosine phosphorylation by AG490 or curcumin led to dose-dependent accumulation of p53 and pRb in cervical cancer cells. Interestingly, the suppression of STAT3 expression or activation was associated with the gradual loss of HPV16 E6 and E7 expression and was accompanied by loss of cell viability. The viability loss was specifically high in HPV16-positive cells as compared to HPV negative C33a cells. These findings substantiate the regulatory role of STAT3 in HPV16-mediated cervical carcinogenesis. Leads obtained from the present study provide a strong rationale for developing novel STAT3-based approaches for therapeutic interventions against HPV infection to control cervical cancer.

## Introduction

Cervical cancer is one of the major women health problem and leading gynecological malignancy of the developing world [1]. India, among other developing countries, is a major contributor to overall cervical cancer prevalence. It contributes disproportionately higher percentage of about 25% of global cervical cancer burden in contrast to about 17% of its contribution to world population. With an annual incidence of 132,000 new cases and mortality rate of 74,000 deaths, cervical cancer is a leading cause of cancer related mortality in Indian women [2]. Among fifteen high-risk human papillomaviruses (HR-HPVs), HPV16 is by far the most dominant and potent type, which is associated with more than 60% of cervical cancer cases globally and upto 90% of the cervical cancer lesions in Indian women [3], [4]. The causal relationship between HR-HPV infection and cervical cancer has become evident from epidemiological and experimental studies [5], [6], [7].

The oncogenic potential of the HR-HPV can be attributed to expression and activity of E6 & E7 [8] whose gene products functionally interfere with the host cell cycle control by interacting with key cell cycle regulators p53 and the retinoblastoma (Rb) proteins, respectively. Expression of HPV E6 and E7 of HPV16 is highly regulated by discrete enhancer elements located on <1 kb length upstream regulatory region, LCR (Long Control Region) that controls activity of P97 promoter and drive transcription from these viral oncogenes and is primarily dependent on host cell factors [9]. Therefore, the expression of these viral oncogenes is controlled by host's sequence-specific ubiquitous and inducible transcription factors. These transcription factors are normally modulated at the level of expression and/or activation by receptors for growth factors, cytokines, hormones and other extracellular signal molecules as well as kinases associated with their downstream signaling [10], [11]. Aberrant expression and activation of both inducible and ubiquitous transcription factors is a common event in carcinogenesis [12]. Several host-cell transcription factors like activator protein-1 (AP-1), nuclear factor kappa B (NF-kB), Sp1, NF-1, TEF-1, TEF-2, Oct-1, AP-2, KRF-1, YY1 and glucocorticoid responsive elements are aberrantly expressed and play a crucial role during development of cervical cancer [13], [14], [15]. Our earlier investigation showed an aberrant expression and constitutive activation of signal transducer and activator of transcription 3 (STAT3) in cervical carcinogenesis that accumulates gradually during the process of cervical carcinogenesis which describes a significant correlation of high risk HPV16 infection in cervical lesions with active STAT3 expression [16]. Presence of defined STAT3 site in the LCR of HPV16 or any other HPV type is not known. Though there are reports of a putative STAT3 binding site at 5's region [14].

In view of the above, in the present investigation we examined functional role of constitutive active STAT3 in regulation of viral oncogenes E6 & E7 and their cellular targets p53 and pRB in HPV16-infected cells. In addition, constitutively active STAT3 was targeted in HPV16 positive cervical cancer cell lines *in vitro* by different STAT3-specific siRNA that knock down STAT3 expression or treatment with STAT3 inhibitors like AG490 and curcumin that block STAT3 phosphorylation to determine the role of active STAT3 in HPV16-mediated cervical carcinogenesis.

## Materials and Methods

### Cell lines and clinical specimens

Established cervical cancer cell lines C33a (HPV-), SiHa and CaSki (HPV16+) cells free of intra/inter species cross-contamination were procured from ATCC and were maintained in prescribed culture conditions. A total of 70 fresh cervical tissue biopsies were collected from 70 malignant cervical tissues prior to any chemo/radio therapy from the Cancer Clinic, Gynae Out Patient Department of Lok Nayak Hospital, New Delhi, India. Written informed consent was obtained from all the subjects included in the study and was carried out in accordance with the principles of the Helsinki Declaration and clinico-epidemiological details were taken from their clinical records. The study was approved by the ethics committee of Institute of Cytology and Preventive Oncology “ICPO Ethical Committee”. A portion of each biopsy collected in cold 1× phosphate buffer saline (PBS) was immediately processed for molecular biological works and the other half was sent for histopathological diagnosis in formalin solution. Out of 70 biopsies examined only 30 could be utilized for analysis of STAT3, pSTAT3, HPV16 E6, E7, p53 and pRB expression studies by immunoblotting and IHC. All reagents used in the study were of analytical or molecular biology grade and procured from Sigma Aldrich (USA) unless specified.

### Cell culture

Cells were maintained in Dulbecco's modified Eagle's medium (DMEM), supplemented with 10% heat- inactivated fetal calf serum and 1% penicillin/streptomycin in CO_2_ incubator with a humidified atmosphere of 95% air and 5% CO_2_ at 37°C.

### STAT3 inhibitors

Commercially available STAT3 siRNA pool containing 3 different siRNA targets to non- overlapping sequences and scrambled siRNA which was used as control were purchased from Santa Cruz Biotechnology (Santa Cruz, CA, USA). Transfection kit used to make transient siRNA transfection was procured from Qiagen, CA, USA. Commercially available curcumin (Sigma, St. Louis, MO, USA) and specific JAK-2 inhibitor, Tyrphostin AG490 (Alexis Biochemicals, San Diego, CA, USA) were freshly dissolved in DMSO and diluted in the medium immediately before use.

### Isolation of total, cytoplasmic and nuclear proteins from cervical tissues and cell lines

Total proteins from freshly collected biopsies and cultured cells were prepared by the method described previously [16] using lysis buffer (20 mM Tris (pH 7.4), 250 mM NaCl, 2 mM EDTA (pH 8.0), 0.1% Triton X-100, 0.01 mg/ml aprotinin, 0.005 mg/ml leupeptin, 0.4 mM PMSF, and 4 mM Na_3_VO_4_. For preparation of cytoplasmic and nuclear proteins, buffer A and buffer B methods were used as described earlier [16]. The concentration of proteins was determined by spectrophotometric method and the proteins were stored in aliquots at −80°C till further use.

### Immunohistochemistry

The immunohistochemical staining was performed as described previously [16]. Briefly, 5 μm section of freshly fixed and paraffinized tissue sections were deparaffinized, rehydrated and subjected to heat-induced epitope retrieval in the 10 mM citrate buffer (pH 6.0). Non-specific binding sites were blocked using 1.5% blocking serum and incubated overnight in pre-standardized dilution of primary antibody. Immunoreactivity was visualized according to manufacturer protocol (ABC staining kit, Santa Cruz Biotech).

### Electrophoretic mobility shift assay (EMSA)

For analysis of STAT3 DNA binding activity after treatment with STAT3 inhibitors in cell lines, nuclear proteins were checked by EMSA. 10 μg of nuclear proteins from each sample were incubated with γ^32^P-radiolabeled oligonulceotide probe containing a hSIE derived from the c-fos gene promoter (sense strand, 5′-AGCTTCATTTCCCGTAAATCCCTA-3′) that binds activated STAT3 proteins. Protein DNA complexes were resolved by non-denaturating PAGE (6%). The gel was dried and detected by Phosphoimager (FLA-5100, Fujifilm, Japan). Quantification of STAT3 activation levels was performed using Alpha Ease FC version 4.1.0 (Alpha Innotech Corporation, IL).

### Immunoblotting

Total cellular proteins (50 μg/lane) were separated in 8–12% polyacrylamide gel and electrotransferred on PVDF membranes (Millipore Corp, Bedford, MA, USA). The membrane was blocked in PBS containing 5% nonfat skimmed milk and probed with specific antibody by incubating the membrane overnight in pre-standardized dilution of primary antibody in blocking solution at 4°C. These blots were washed, incubated with HRP-antimouse IgG secondary antibodies and visualized by Luminol detection kit (Santa Cruz Biotech, USA) and by exposing the blot to KODAK X-Omat films (Kodak India, India). The western blot membranes were reprobed for β-actin expression as an internal control. The quantitative densitometric analysis of the bands was performed using Alpha Ease FC version 4.1.0 (Alpha Innotech Corporation, IL).

### Transient transfection

For STAT3 RNA interference assay, 2×10^5^ cells per well in a six well tissue culture plate were seeded in 2 ml antibiotics-free normal growth medium supplemented with 10% FBS and incubated at 37°C in a CO_2_ incubator for 18–24 h until the cells are 30–40% confluent. Cell were exposed to RNAiFect transfection reagent (Qiagen) in the presence of 10 nM siRNA against STAT3 (Santa Cruz) or scrambled siRNA (Control) according to the manufacturer's instruction. Cells were then incubated for 48 h, followed by microscopic examination and protein extraction.

### MTT assay

The cytotoxic effects of curcumin and AG490 against SiHa and C33a cells were determined by MTT dye uptake method [17]. The cells were incubated in triplicate in a 96-well plate in the presence or absence of indicated test samples in a final volume of 0.1 ml for 24 h at 37°C in a CO_2_ incubator. Thereafter 0.025 ml of MTT solution (5 mg/ml in PBS) was added to each well. After 2 h incubation at 37°C, lysis buffer (20% SDS and 50% Dimethyl Formamide) was added, and the extract was incubated overnight at 37°C for solublization of formazan crystals. The OD at 570 nm was measured using a 96-well multiscanner autoreader (Biotek, Winooski, Vermont) with the lysis buffer serving as blank. The percentage of cell viability was calculated using the following formula: Percentage cell viability  =  (OD of the experiment samples/OD of the control) ×100. IC_50_ was determined as described earlier.

### Flow cytometric analysis of apoptotic cell death by Annexin V-FITC

Cells were treated with curcumin (50 µM) and AG490 (100 µM) for 24 h. The cells were then harvested, washed with PBS and incubated with AnnexinV-conjugated fluorescein isothiocynate (FITC) and propidium iodide (PI) for cellular staining as described in AnnexinV-FITC apoptosis detection kit (BD Biosciences) manufacturer's instructions. The stained cells were then analyzed by FACS. The number of 10000 events was acquired and the cells were properly gated for analysis using FACSAria instrument equipped with Flowjo software (Becton-Dickinson Biosciences, San Jose, CA).

### Quantitation of caspase-3 activity

The activity of caspase-3 was measured using the active caspase-3 apoptosis kit (BD Pharmingen, USA) following the manufacturer's protocol. Briefly, cells were treated with curcumin (50 µM) and AG490 (100 µM) for 24 h and were harvested by pooling attached and detached cells were pelleted with centrifugation at 200×g for 5 min at 4°C. The cells were permeabilized, fixed, and stained for active caspase-3 (PE-conjugated) as described in manufacturer's protocol (BD Biosciences).

### Measurement of mitochondrial membrane potential

SiHa cells were plated onto a 60-mm tissue culture plate at subconfluent density. After 24 h incubation cells were treated with curcumin (50 µM) and AG490 (100 µM). After 24 h cells were incubated with 5 μM JC-1 fluorescence dye for 30 min in CO_2_ incubator and washed several times with PBS pre-warmed at 37°C. Mitochondrial membrane potential was evaluated qualitatively under a fluorescence microscope (Olympus IX81) using 568 nm filter.

### Statistical analysis

The data analysis was performed using the statistical software SPSS version 17 or Sigma Stat Graph Pad Instant (version 4.0). Two-tailed Fisher's Exact test was used to compare the expression of proteins among different histopathological grades of tissue biopsies. *p* values of <0.05 were considered statistically significant.

## Results

### Association of constitutively active STAT3 with expression of HPV16 E6 & E7 and their respective cellular targets, p53 & pRB in HPV16-positive cervical cancer cells

**Figure 1:**
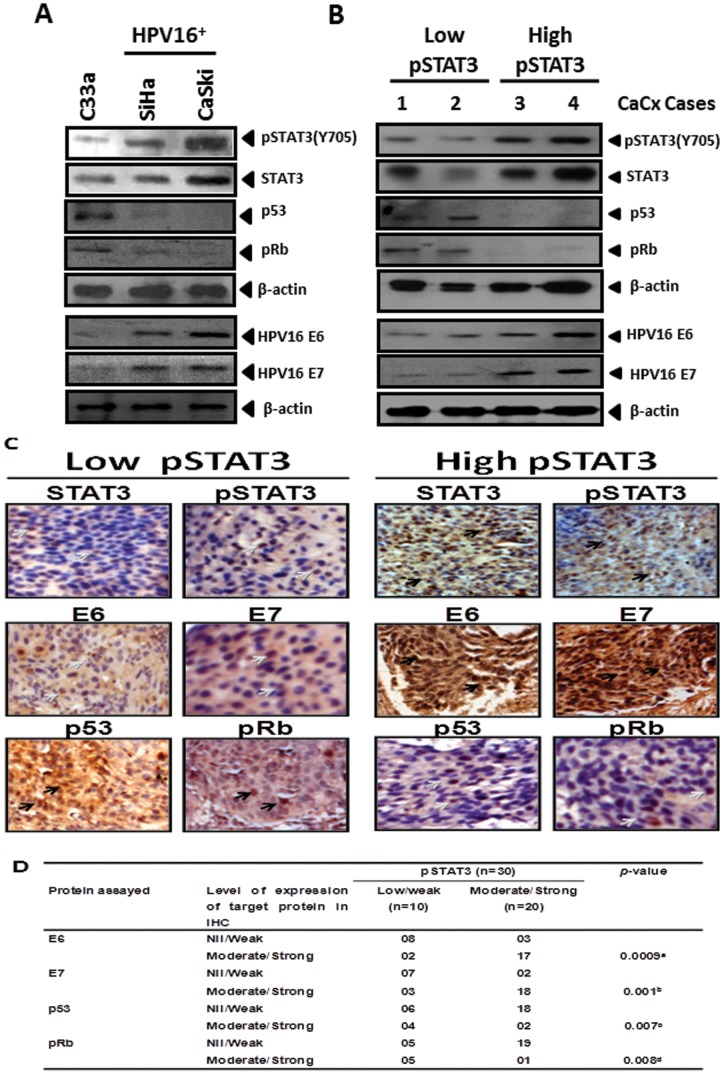
Increased STAT3 activity in cervical cancer cells is associated with elevated expression of HPV16 E6 & E7 and corresponding loss of expression of their respective cellular targets, p53 and pRb. (**A**) Representative immunoblots indicating differential expression of active pSTAT3 (Y705), STAT3, HPV16 E6 & E7, p53 and pRb in HPV negative (C33a), and HPV16 positive (SiHa & Caski) cervical cancer cell lines. (**B**) Expression of HPV16 E6, E7, p53 and pRb proteins in representative cervical cancer cases (#1–4) with differential pSTAT3/STAT3 expression [low (Case #1 and #2) vs. high (Case #3 and #4)]. Total cellular proteins (50 µg) isolated from C33a, SiHa and Caski cells (1×10^6^ cells) and cervical cancer tissues were resolved on 10 or 15% SDS-PAGE and immunoblotted for phospho-STAT3, STAT3, HPV16 E6, HPV16 E7, p53 and pRb proteins by specific antibodies as described in ‘Methods’. Blots were stripped and reprobed for β-actin as loading control. (**C**) Representative photomicrographs of immunohistochemical analysis of STAT3, pSTAT3 (Y705), HPV16 E6, E7, cellular p53 and pRb in cancer lesions with differential pSTAT3/STAT3 expression. Freshly fixed, paraffin-embedded sections (5 μm) of cervical tissues from cancerous lesion of the cervix were processed for IHC and probed for indicated antibodies and detected by HRP-DAB as described in ‘Methods’. Brown precipitate indicates immune reactive-positive cells, blue stain represents nuclei. Black and grey arrows indicate strong and weak immunopositive cells respectively. (Original magnification: 200×). (**D**) Cumulative immunohistochemical data indicating association of level of active STAT3 with expression of HPV16 oncogenes E6 and E7 and their cellular targets p53 and pRb. Arbitrary staining intensity grades of respective proteins in immunohistochemistry were categorized into Strong, Moderate, Weak or Nil/not detectable as described in ‘Methods. Values indicate the distribution of specimens in each category. ^a^ *p* value between pSTAT3 nil/weak or Moderate/Strong versus HPV16 E6 nil/weak or moderate/strong; ^b^ *p* value between pSTAT3 nil/weak or Moderate/Strong versus HPV16 E7 nil/weak or moderate/strong; ^c^ *p* value between pSTAT3 nil/weak or Moderate/Strong versus p53 nil/weak or moderate/strong; ^d^ *p* value between pSTAT3 nil/weak or Moderate/Strong versus pRb nil/weak or moderate/strong, as determined by two tailed Fischer’s Exact Test.

To determine the functional contribution of constitutively active STAT3, we first examined the expression of HPV16 E6, E7, p53 and pRB and correlated with STAT3 expression and its activation in both, cervical cancer cell lines, and cervical tumor tissues. Immunoblotting analysis of cellular proteins was performed to assess the expression of pSTAT3 (Y705), STAT3, HPV16 E6, E7 and cellular p53 and pRB proteins in SiHa, CaSki, and C33a cells. The results showed differential expression pattern of constitutively active pSTAT3 (Y705) among the cell lines analyzed. In comparison to the HPV-negative C33a cells, HPV16-positives SiHa and Caski cells demonstrated higher level of pSTAT3 and STAT3 expression (**Fig. 1A**). These cells also revealed a specific expression of HPV16 E6 and E7 which was corroborated with high STAT3 and pSTAT3 expression, whereas E6 and E7 expression was completely absent in HPV negative C33a cells thus confirming specific binding of the antibody to respective proteins and indicated existence of a potential association between expression and activation of STAT3 and E6/E7 expression in cervical cancer cell lines (**Fig. 1A**). When these cell lines were tested for p53 and the hypophyosphorylated pRB protein using specific antibodies that detected N terminal region of p53 or antibody that specifically detected unphosphorylated pocket region of pRB located between 514–610 residues, the expression of p53 and pRB was detected at extremely low level or was undetectable in HPV16 positive cells. In contrast, both p53 and pRB proteins were expressed at significantly higher levels in C33a cells.

Further, we analyzed E6, E7, p53 and pRB expression by immunoblotting and immunohistochemistry, in 30 cervical cancer specimens with pre-defined status of pSTAT3 (Y705)/STAT3 expression. Ten out of 30 (33%) specimen had low levels of pSTAT3/STAT3 whereas 20/30 (67%) specimen had moderate or high pSTAT3/STAT3 expression. As shown in representative immunoblots in **Fig. 1B**, cervical cancer tissues having low level of activated STAT3 were having correspondingly low levels of HPV16 E6 and similar low levels of HPV16 E7 protein expression. On the contrary, these samples demonstrated detectable level of p53 and pRB proteins. On the other hand, lesions that expressed moderate or high levels of pSTAT3/STAT3 expressed stronger HPV16 E6 and E7 bands in immunoblot analysis reflecting high levels of E6 and E7 proteins. Immunoblots performed on cellular proteins of these lesions, however, did not detect any p53 and pRB. These findings were corroborated with immunohistochemical data presented in **Fig. 1C**, which show an expression dynamics of STAT3, pSTAT3 that matched with expression pattern of E6 and E7 with a reciprocal reduced expression of p53 and pRB in majority of cancer lesions. Statistical analysis revealed a significant positive correlation (p<0.05) between the expression level of pSTAT3 with expression of viral oncoprotein E6 & E7 and an inverse correlation with their downstream targets, p53 and pRB (**Fig. 1D**).

### Functional significance of STAT3 in regulation of HPV16 viral oncogene expression: RNA interference of STAT3 decreases cell viability and induce growth inhibition in cervical cancer cells

**Figure 2:**
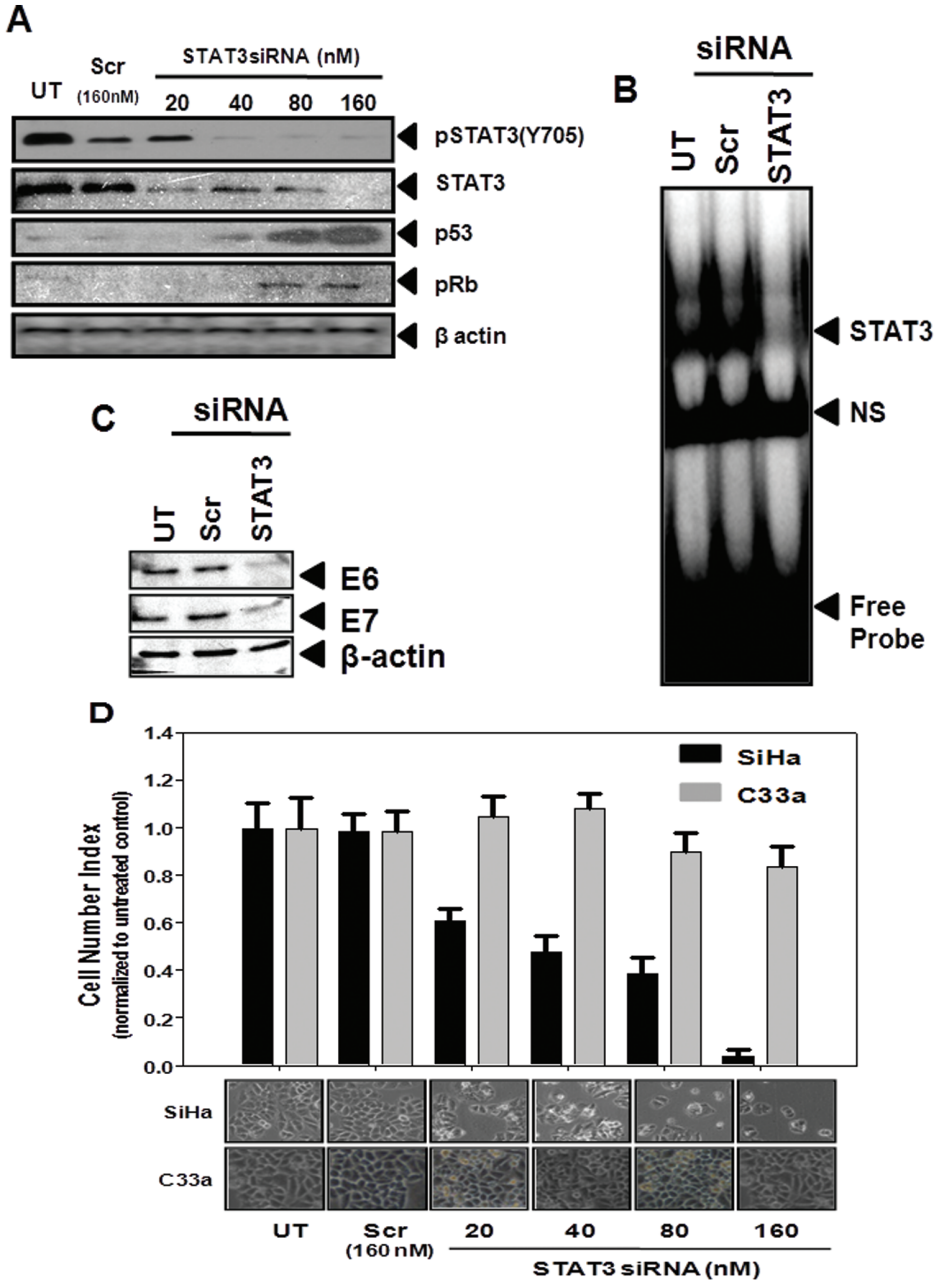
Effect of targeting STAT3 expression by RNA interference on the expression of p53 & pRb and E6 & E7 and on viability of cervical cancer cells. (A) Specific STAT3 siRNA inhibits STAT3 and pSTAT3 expression in cervical cancer cells. Cellular proteins (50 µg) isolated from SiHa cells (2×10^5^ cells) treated with the indicated concentrations of STAT3 and control siRNA (Scr) for 48 h were examined for pSTAT3 (Y705), STAT3, p53, or pRb by immunoblotting as described in ‘Methods’. Blots were stripped and reprobed with β-actin as loading control. (B) Nuclear proteins (10 µg/lane) isolated from SiHa cells treated with STAT3 or control siRNA were incubated with radiolabeled STAT3 oligos to check STAT3-specific DNA binding activity by EMSA. NS- Non specific band. (C) Blocking STAT3 by specific siRNA abrogates expression of HPV16 oncogenes E6 and E7 in cervical cancer cells. Cellular proteins isolated from SiHa cells (2×10^5^ cells) treated with the STAT3 and control siRNA (160 nM) for 48 h were examined for expression of HPV16 oncogenes E6 and E7 by immunoblotting. Blots were stripped and reprobed with β-actin as loading control. (D) STAT3 inhibition was accompanied with reduced proliferation/ loss of cell viability in HPV16 positive cervical cancer cells. HPV16 positive SiHa cells and HPV negative C33a cells (2×10^5^ cells) treated with the indicated concentration of STAT3-specific and control siRNA for 48 h were harvested by trypsinization and counted for live cells using Trypan blue vital dye. Experiment was performed in triplicate, error bars indicates mean±SD. Lower panel indicates photomicrographs of respective control and treated SiHa and C33a cell cultures prior to their harvesting for cell counting.

To test the functional relevance of active STAT3 in the expression of HPV16 oncogenes we performed *in vitro* silencing of STAT3 in cervical cancer cells using siRNA against STAT3. As shown in **Fig. 2A**, transfection of SiHa cells by increasing concentration of STAT3 siRNA resulted in a dose-dependent decline in expression level of STAT3, whereas, STAT3 expression remained unaltered in scrambled siRNA-treated control cells. Inhibition of STAT3 expression was observed at concentrations as low as 20 nM and was completely abolished at 160 nM. Interestingly, the reduction of STAT3 expression was accompanied with a concomitant reduction in signal intensity of pSTAT3 more rapidly than STAT3 which indicated a specific effect of siRNA-mediated inhibition on STAT3 expression and its activity. Nuclear proteins isolated from these siRNA-treated cells were also examined to test their STAT3-DNA binding activity by EMSA which revealed absence of STAT3-specific DNA binding activity in siRNA-treated cells whereas there was no effect on STAT3 binding in control cells treated with scrambled siRNA (**Fig. 2B**).

Following establishment of STAT3-specific silencing in cervical cancer cells, cellular proteins derived from siRNA-treated SiHa cells were examined by immunoblot analysis for p53 and pRB expression to study the cellular effects of STAT3 silencing. Interestingly, increasing dose of STAT3 siRNA that abrogated STAT3 expression resulted in a dose-dependent accumulation of p53 and pRB proteins in these treated SiHa cells (**Fig. 2A**). Both p53 and pRB were detectable in cells treated with as low as 40 nM of STAT3 siRNA and accumulation of these proteins increased further and was maximal at 160 nM. In contrast, control cells treated with scrambled siRNA failed to show any expression/accumulation of either p53 or pRB protein.

Specific effects of STAT3 silencing that resulted in accumulation of p53 and pRB were also examined on expression of viral oncoproteins E6 and E7 that showed an association with high levels of pSTAT3 in cervical cancer lesions. To analyze this proposition, we checked the expression of HPV16 viral oncogenes, E6 and E7, in cellular proteins derived from siRNA-treated SiHa cells by immunoblotting. As shown in **Fig. 2C** transfection with STAT3 siRNA at 160 nM concentration resulted into >90% loss of E6 and E7 expression in SiHa cells, whereas it was unchanged in cells treated with equi-molar concentration of scrambled control siRNA.

Simultaneously, with the analysis of oncogenic mediators E6 and E7, and tumor suppressor gene, p53 and pRB, we examined effect of STAT3 silencing on overall cell growth by enumerating the number of cells harvested following 48 h treatment of cells with scrambled control and STAT3-specific siRNA. To study the STAT3-specific effect on HPV16 positive SiHa cells, C33a cells which expressed significantly lower levels of STAT3 and lacked HPV infection and associated oncogenic signaling were similarly treated for comparison. As shown in photomicrographs taken prior to harvesting and counting of the treated cells and depicted in **Fig. 2D**, there was a dose-dependent reduction in cell proliferation of SiHa cells treated with STAT3 siRNA, whereas similarly treated C33a cells were only marginally affected at doses 80 nM or higher. These observations directly reflected on results obtained from counting of these treated cells following trypsinization. SiHa cells treated with 80 and 160 nM of STAT3-specific siRNA resulted in reduction of more than 60% and 95% of cells, respectively as compared to the control or scrambled-treated cultures.

### Pharmacological targeting of STAT3 by curcumin and tyrphostin AG490 inhibit constitutive active STAT3 by blocking STAT3 DNA binding activity

**Figure 3:**
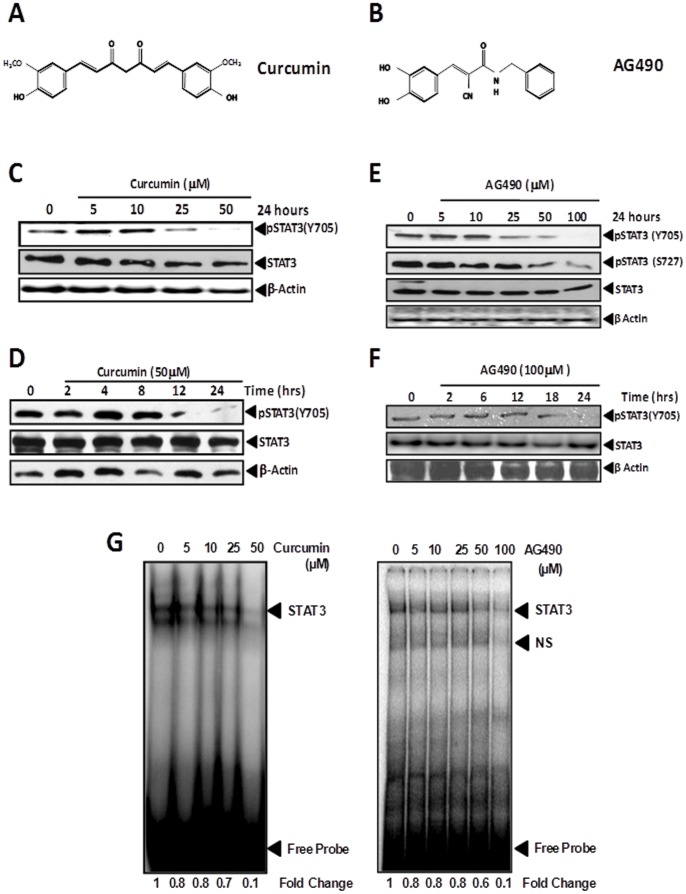
Effect of curcumin and AG490 on STAT3 expression and phosphorylation in cervical cancer cells. Chemical structure of curcumin (**A**) and AG490 (**B**). Curcumin and AG490 inhibit constitutive STAT3 phosphorylation in cervical cancer cells. (**C, E**) Dose-dependent inhibition of STAT3 phosphorylation by curcumin (**C**) and by AG490 (**E**). SiHa cells (1×10^6^ cells) treated with indicated concentration of curcumin and AG490 for 24 h were tested for expression of phospho-STAT3 by immunoblotting. Subsequently the blots were stripped and reprobed with STAT3 and β-actin. (**C, E**) Time kinetics of inhibition of STAT3 phosphorylation in curcumin- (**D**) and AG490- (**F**) treated cervical cancer cells. SiHa cells (1×10^6^ cells) treated with curcumin (50 µM) or AG490 (100 µM) for indicated durations were tested for expression of phospho-STAT3 by immunoblotting. Subsequently the blots were stripped and reprobed with STAT3 and β-actin. (**G**) Downregulation of STAT3 phosphorylation by curcumin and AG490 is associated with abrogation of STAT3 DNA binding activity. Nuclear proteins (10 μg) isolated from SiHa cells (1×10^6^ cells) treated with the indicated concentrations of curcumin (*left panel*), or with AG490 (*right panel*) for 24 h were incubated with specific hSIE probes, assayed for STAT3 binding by EMSA as described and intensities of STAT3-specific band quantified are indicated. NS- Non-specific bands.

Apart from aberrant/overexpression, STAT3 is extensively phosphorylated in cervical carcinogenesis indicating persistence of upstream activation signals. Therefore, in addition to targeting the expression of STAT3, in the next part of the investigation, we attempted to block its activity by curcumin and tyrphostin AG490. Curcumin (**Fig. 3A**) is known to inhibit STAT3 phosphorylation by EGFR, Src, and Jak2, the upstream kinases responsible for activation of STAT3 whereas AG490 (**Fig. 3B**) is a specific inhibitor of Jak2 kinase which is primarily responsible for STAT3 phosphorylation through IL-6 receptor. In view of the above, we treated SiHa cells with different concentrations of curcumin and AG490, and checked for the expression and phosphorylation of STAT3 in these cells. As shown in **Fig. 3C** **,** immunoblot analysis revealed decline in expression level of phospho-STAT3 (Y705) in a dose-dependent manner. The significant decline in the pSTAT3 level was apparent in cells treated with 25 µM curcumin, whereas, it was completely abolished in cells treated with 50 µM curcumin. Interestingly, STAT3 (un-phosphorylated) levels remained unchanged in these cells irrespective of the curcumin concentration indicating specific-inhibition of STAT3 phosphorylation. Further, we analyzed time-course of the inhibitory effect of curcumin on STAT3 phosphorylation. **Fig. 3D** demonstrate a time-dependent decline in STAT3 phosphorylation in cells treated with increasing duration of curcumin treatment which was observable after 12 h of treatment and was maximal by 24 h.

SiHa cells were similarly treated with increasing concentrations of AG490 and were analyzed for STAT3 and pSTAT3 expression. As shown in **Fig. 3E**, the immunoblot analysis revealed a dose-dependent decline in expression levels of phospho-STAT3 (Y705), whereas overall expression of STAT3 remained unaltered in AG490-treated cells. Inhibition of pSTAT3 (Y705) phosphorylation was observable in cells treated with 25 µM, whereas, treatment of cells at 100 µM AG490 resulted in complete loss of phosphorylation at the tyrosine residue 705, for which this inhibitor is specifically designed. However, at such higher concentrations, AG490 treatment resulted in partial decline of pSTAT3 (S727) levels indicating a non-specific inhibitory effect on serine phosphorylation (**Fig. 3E**). Study of the time course of AG490's inhibitory effect on tyrosine phosphorylation in SiHa cells revealed a delayed effect as compared to curcumin as cells treated with AG490 for 12 h did not show any alteration in pSTAT3 expression, and its level started declining by 18h and was completely abolished by 24 h of AG490 treatment (**Fig. 3F**).

Further, to determine the functional significance of inhibitory effect of curcumin and AG490 on STAT3 phosphorylation, the treated cells were evaluated by assessing STAT3-specific DNA binding activity in SiHa cells. As shown in **Fig. 3G** *(left panel)*, curcumin effectively blocked the STAT3 DNA binding in a dose dependent manner. Cell treated with curcumin (50 µM) resulted in loss of more than 90% of the activity as compared to the controls. On the other hand similar degree of inhibition was observed in cells treated with 100 µM AG490 (**Fig. 3G,** *right panel*).

### Effect of curcumin- and AG490-induced inhibition of constitutively active STAT3 on cellular p53 and pRB and viral E6 and E7 oncoprotiens

**Figure 4:**
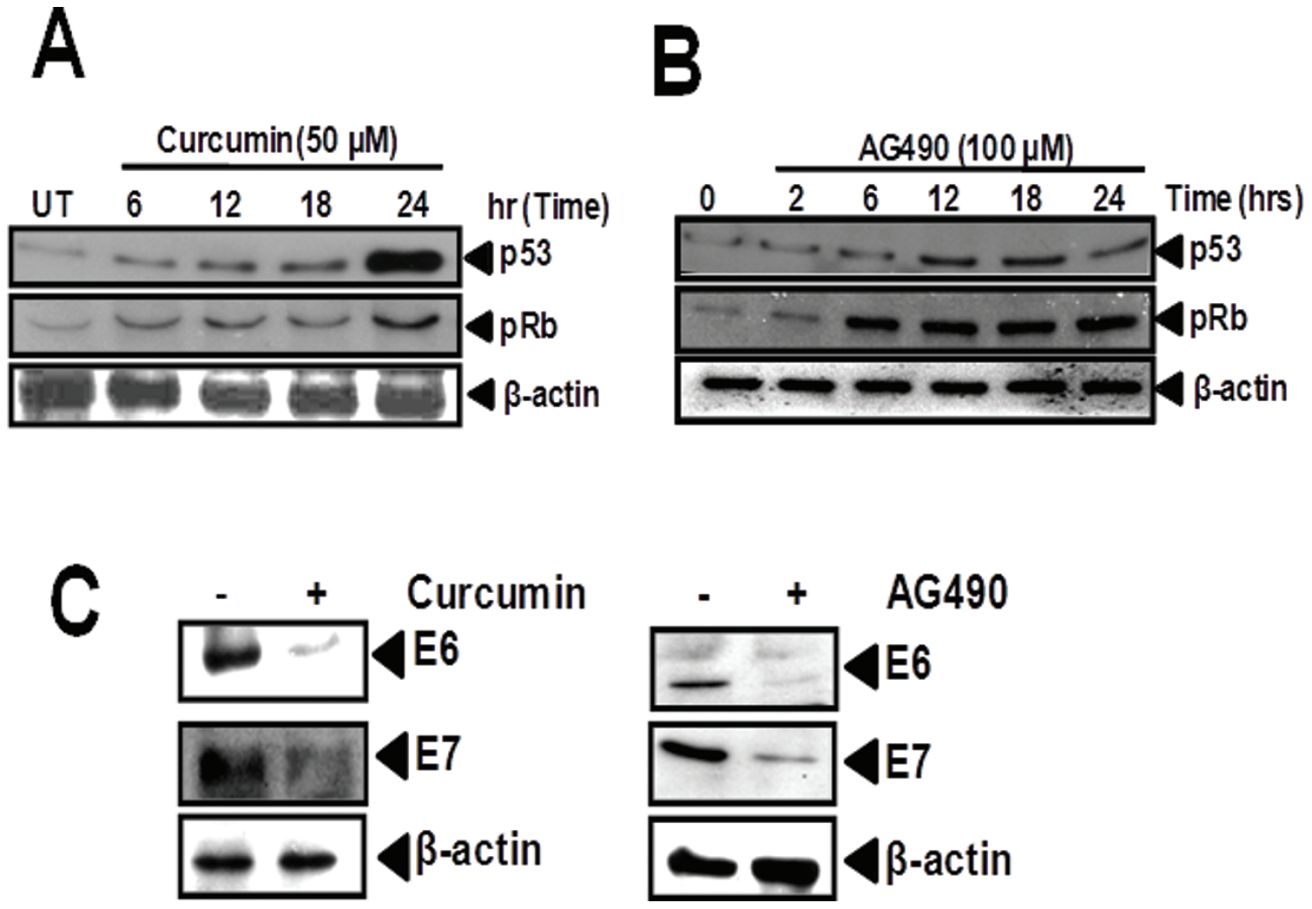
Both, curcumin and AG490, rescue expression of p53 and pRb through suppression of HPV16 E6 and E7 expression. (**A–B**) Effect of curcumin and AG490 on p53, pRb expression in cervical cancer cells. SiHa cells (1×10^6^ cells) treated with the curcumin (50 µM) or AG490 (100 μM) for indicated durations were tested for p53 and pRb expression by western blotting as described. (**C**) Curcumin and AG490-mediated abrogation of HPV16 E6 and E7 expression. SiHa cells (1×10^6^ cells) treated with curcumin (50 µM) or AG490 (100 μM) for 24 h were tested for HPV16 E6 and E7 expression by immunoblotting as described. Blots were stripped and re-probed with β-actin as loading control.

In the next part of our investigation, we examined the effect of curcumin and AG490-mediated inhibition of STAT3 phosphorylation on expression of cellular p53 and pRB and viral E6 and E7. Curcumin at concentration of 50 µM and within 12 h was found to upregulate p53 level by 2 fold compared to untreated controls, whereas upto 5 fold increase in p53 level was observed by 24 h (**Fig. 4A & B**). Accumulation of hypophosphorylated form of pRB was also observed upto 3.5 fold in cells treated with curcumin for 24 h. On the other hand, 100 µM of AG490 induced p53 accumulation similar to curcumin-treated cells, however, hypophosphorylated form of pRB by AG490 was found at much higher levels and was more than 4 fold by 6 h and was consistently maintained at high levels till 24 h (**Fig. 4B**). The p53 expression and pRB accumulation was found selective since expression of other housekeeping gene, i.e. β-actin remained unaffected. Curcumin and AG490 treated cells were also examined for expression of HPV16 E6/E7 oncoprotein levels in curcumin-treated SiHa cells. Immunoblotting for E6 and E7 proteins demonstrated a significant decline in the levels of E6 and E7 proteins in cells treated with curcumin as well as AG490 for 24 h (**Fig. 4C**).

### Effect of curcumin- and AG490 on cell growth and viability of cervical cancer cells

**Figure 5:**
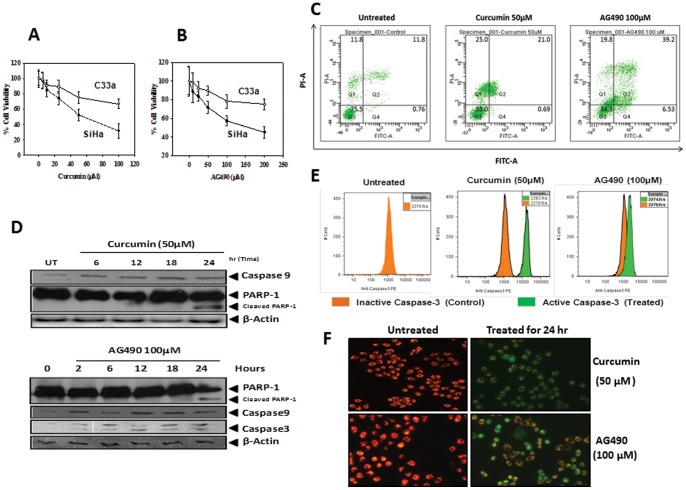
Effect of curcumin and AG490 on growth and survival of cervical cancer cells. **A&B.** Curcumin and AG490 demonstrate strong cytotoxic effect on HPV16 positive cervical cancer cells. Cell viability of HPV16 positive SiHa and HPV negative C33a cells treated in triplicates with indicated doses of curcumin (**A**) and AG490 (**B**) for 24 h was measured by MTT assay as described in “Methods”. Error bars indicates mean±SD. **C–F**. Curcumin and AG490 induce apoptosis in cervical cancer cells. Flowcytometric evaluation of AnnexinV-PI stained SiHa cells incubated in the absence or presence of curcumin (50 µM) and AG490 (100 µM) for 24 h (**C**). Tabulated data indicates percentage of cells in different apoptotic stages of a representative experiment. (**D**) Treatment of curcumin and AG490 induce activation of caspase-3 and cleavage of its downstream substrate PARP-1. Cellular proteins (50 μg) prepared from SiHa cells treated with curcumin (50 µM) and AG490 (100 µM) for the indicated times were examined for expression of caspase-3 and PARP by immunoblotting. (**E**) Flowcytometric evaluation of permeabilized, fixed, and active caspase-3-stained SiHa cells incubated in the absence or presence of curcumin (50 µM) and AG490 (100 µM) for 24 h. (**F**) Curcumin and AG490 exposure results in changes in the mitochondrial membrane potential. Fluorescence photomicrographs of SiHa cells treated with curcumin (50 µM) or AG490 (100 µM) for 24 h and loaded with JC-1 stain were examined for fluorescence on green and red channel. Photographs represent overlay of red and green signals. Cells appearing in red channel indicate intact mitochondrial transmembrane potential whereas green indicate loss of mitochondrial membrane potential.

To analyze the consequences of inhibition of constitutively active STAT3 by curcumin and AG490 in cervical cancer cells, SiHa cells were treated with increasing concentrations of curcumin and AG490 for 24 h and their cell viability was checked by MTT assay. To assess HPV16-specific effects, HPV negative C33a cells were similarly treated and the viability was measured. A comparison of cell viability in curcumin-treated SiHa and C33a cells revealed a 50% cell growth inhibition (IC_50_) at 50 µM in SiHa cells. In contrast, at this curcumin concentration, C33a cells showed only ∼25% inhibition and, thus, were found resistant to curcumin (**Fig. 5A**). Similarly, SiHa and C33a cells treated with AG490 revealed a differential effect on cell growth and viability. SiHa cells were found more susceptible to AG490-induced growth inhibition which could be attributed to presence of HPV16 in these cells (**Fig. 5B**). As MTT assay measures the mitochondrial activity in the cultures and cannot differentiate inhibition of cell proliferation from loss of cell viability, we examined curcumin and AG490-treated SiHa cells using flowcytometry by staining with Annexin V-propidium iodide (PI) which distinguishes live cells from the cells undergoing apoptosis or necrosis. The four quadrant analysis of curcumin and AG490-treated SiHa cells depicted in **Fig. 5C** revealed positive staining of Annexin V demonstrating presence of phosphotidyl serine on outer membrane and compromised membranes resulting in PI-positive staining in most of the treated cells, a hallmark feature of late apoptosis, in 24 h treated cultures. In coherence with the MTT results, the proportion of cells undergoing apoptosis was considerably higher in curcumin- treated cultures. Curcumin and AG490-treated cells were also checked for expression and activity of caspase-3, its upstream activator caspase-9 and downstream substrate, Poly [ADP-ribose] polymerase 1 (PARP-1) in treated cells. As indicated in **Fig. 5D**, treatment of SiHa cells with 50 µM curcumin for 24 h induced the expression of caspase-9 and appearance of 85 kDa cleavage product of PARP-1. Similarly, AG490 (100 µM) treatment also resulted in induction of caspase-9, cleavage of procaspase-3 into 20 kd cleavage product by 6 h and a consequent proteolytic cleavage of PARP-1 which was partially cleaved and was maximum at 24 h.

It was interesting to note that SiHa cells treated with curcumin or AG490 did not induce prominent cleavage of PARP-1, its canonical substrate, and majority of it remained intact which made us to investigate the specific presence of active caspase-3. Flowcytometric analysis of cells revealed a considerable proportion of cells expressing the active caspase-3, both in curcumin as well as in AG490-treated cultures which corroborated well with the data obtained from AnnexinV-PI staining (**Fig. 5E**). To further substantiate our observation regarding induction of apoptosis in cervical cancer cells by curcumin and AG490, the treated cells were subjected to JC-1 staining which is used to assess the integrity of the mitochondrial membrane potential and stains the mitochondria red when their membranes are intact and polarized and give green fluorescence when these membranes are depolarized. Fluorescence microscopic evaluation of JC-1-stained untreated SiHa cells showed intact red-stained mitochondria whereas cells treated with curcumin or AG490 revealed green staining indicating loss of mitochondrial membrane potential (**Fig. 5F**).

## Discussion

It is well established that LCR plays a central role in recruitment of cellular transcription factors and induce expression of viral oncogenes, E6 and E7 through its action of p97 promoter. These viral oncogenes are responsible for cellular transformation by targeting critical cell cycle regulators, p53 and pRB of the host cells [18], [19], [20]. However, the role of STAT3 in expression of HPV oncogenes and their cellular targets in cervical carcinogenesis is not known. Our results revealed a positive correlation of active STAT3 with E6 and E7 and an inverse relation with p53 and pRB pools. Immunoblotting analysis of cellular proteins from HPV16 positive and HPV negative cell lines revealed high expression of pSTAT3, E6 and E7 in HPV16 positive, SiHa and CaSki cells which were inversely correlated with low levels of p53 and hypophosphorylated pRB. These preliminary observations when examined in tumor biopsies with differential pSTAT3 levels revealed that cervical lesions with a moderate or high level of active pSTAT3 demonstrated correspondingly high levels of HPV16 E6 and E7 expression whereas expression of p53 and pRB proteins was undetectable in majority of these lesions. On the other hand, cancer lesions having low levels of pSTAT3 expressed low levels of E6 and E7 and had correspondingly high p53 and pRB expression in such lesions. Despite viral etiology and necessity of viral persistence in cervical cancer, preliminary studies reporting active STAT3 in cervical pre-cancer and cancer lesions [21], [22], [23], [24] incidentally did not correlate it with any of the parameter of HPV infection due to which interaction between these two important arms of cervical carcinogenesis remained unexplored. Moreover, a variable level of viral E6, E7 as well as p53 and pRB levels were reported in several studies [25], [26], [27], [28], [29], [30], [31], though the reasons for such variability were not known. Recent studies indicate that even though viral load (E6/E7 DNA) also increases with progression of cervical lesions, levels of HPV16 E6 and E7 transcripts that increase in progressive lesions are more relevant in disease prognosis [31], [32], [33]. It is important to note that levels of E6/E7 transcript are direct indicators of active transcription as half life of these transcripts is about 2 h. [34]. Our data indicate that STAT3 could be one of the set of transcription factors whose activity might be playing a major role in expression of these viral transcripts. Variations in STAT3 expression and activity along with similar variations in other inducible transcription factors like AP-1 and NF-κB; or ubiquitous transcription factors like SP-1, NF-1, YY1 or Oct-1 may be responsible for variable expression of viral genes/oncogenes which subsequently affect cellular p53 and pRB pools. Indeed, when we analyzed intra-lesional E6 and E7 levels along with p53 and pRB protein in cancer cases with differentially expressing pSTAT3, a significant correlation between pSTAT3 and E6 and E7 levels was observed.

In the next part of our investigation, we examined the functional relevance of correlation between E6/E7 expression and STAT3 in HPV16-induced cervical carcinogenesis by evaluating the effect of specific silencing of active STAT3 either by targeting its expression using specific siRNA or targeting its tyrosine phosphorylation which is essential for its translocation into the nucleus and subsequent DNA-binding activity, by known inhibitors, curcumin and AG490 on expression of p53, pRB and E6 and E7 in an *in vitro* system. Transient transfection of HPV16 positive SiHa cell with STAT3 siRNA which induced in dose-dependent decline in expression of STAT3 and pSTAT3 and decreased DNA binding activity of STAT3 resulted in accumulation of p53 and pRB with a concomitant loss of HPV16 E6 and E7 oncogene expression. Similar, effect on E6, E7, p53 and pRB was observed when pSTAT3 was targeted specifically to inhibit its Tyr705 phosphorylation by curcumin or AG490 that abolished its DNA-binding activity. Suppression of E6/E7 expression and appearance of p53 and pRB in cells with either transiently silenced STAT3 or cells treated with curcumin or AG490 was accompanied by growth inhibition of specifically and strongly of the HPV16-positive cervical cancer cells. In comparison, growth properties of similarly treated HPV negative cells were less affected, thus indicating a specific effect of STAT3 silencing on HPV16 cells via inhibition of E6 and E7 expression. Specific targeting of STAT3 expression in cervical cancer cell lines have been performed earlier using recombinant adenoviral dominant negative STAT3 [21] or STAT3 specific siRNA [22] which invariably demonstrated similar decrease in cell numbers and affected the viability of cervical cancer cells. Interestingly, specific silencing of E6/E7 using specific siRNA also results in similar growth inhibition of cervical cancer cells, loss of transformed phenotype, induce apoptosis and replicative senescence and inhibited tumor formation in animal models [35], [36], [37], [38], [39], [40], [41]. The most interesting feature of E6 and E7 targeting by siRNA is the stabilization of E6 target protein p53 and its nuclear accumulation which eventually leads to the expression of p21 (WAF1/CIP1) [38], [39], appearance of hypophosphorylated RB protein (pRB 105) similarly pharmacological targeting of E6/E7 resulted in accumulation of p53 and was accompanied by reduced STAT3 phosphorylation [42]. Moreover, delivery of E6/E7 siRNA into nude mice has shown significant reduction in the number of tumor nodules and retarded tumor growth of HPV16 positive cells in NOD/SCID mice [38], [39]. These observations, therefore, strongly support inhibition of HPV16 E6 and E7 transcription by targeting STAT3 expression.

Though we observed elevated levels of p53 and pRB in cells with abrogated/targeted STAT3, our results do not claim that loss of expression of oncoproteins E6/E7 is the direct and sole cause of p53/pRB accumulation in cervical cancer cells. STAT3-mediated reciprocal regulation of p53 and pRB expression is already a well-known fact [43], [44]. STAT3 regulate expression and function of p53 by binding to the p53 promoter and resulting in decreased de novo expression of p53 and manifestation of a phenotype similar to the p53 mutants [43]. STAT3 activity also influences p53 response genes and prevents the p53-mediated tumor cell apoptosis. Moreover, STAT3 has been shown to be critically required for full neoplastic transformation by the large Tumor Antigen, TAg [44]. Blocking of binding of TAg to pRB thereby inhibiting the inactivation of pRB results in reduced STAT3 activation whereas genetic ablation of pRB increase the STAT3 activity. In addition, STAT3 directly regulates Cyclin D1 which in turn is a key regulator of phosphorylation of Rb [45]. On the other hand, increased level of p53 is reported to enhance hypophosphorylated form of Rb [46]. These points explain direct effect of reduced STAT3 on p53 and pRb upregulation. Therefore, preventing expression of E6/E7 through STAT3 inhibition in cervical cells could only partially contribute to development of p53/pRB pools which may otherwise get independently up-regulated through de novo synthesis as a result of lowering of the active STAT3 levels.

Interestingly, the reduction of STAT3 expression was accompanied with a concomitant reduction in pSTAT3 levels. Once activated in malignant cell, STAT3 can induce expression of a subset of genes which are further important for STAT3 activation and are responsible for maintaining its sustained activity [47]. Activated STAT3 induces expression of several genes including IL-6, IL-22, EGF, IL-23 and IL-10 [48], [49] as well as induction of cell surface growth factor and cytokine receptors (EGFR, c-Met, IL-23R) or cytoplasmic protooncogenes such as K-Ras, Src and c-Abl, whose products are capable of inducing STAT3 phosphorylation [50], [51], [52], [53]. On the contrary, persistent STAT3 activation also checks its negative regulator PTEN which is responsible for its dephosphorylation [54] by inducing specific regulatory microRNA, miR-21 that is found elevated in cervical cancer cells [55], [56]. It is likely that targeting STAT3 rapidly relieve miR-21-mediated suppression resulting in appearance of PTEN-induced dephosphorylation. Similar to PTEN, other negative regulators of STAT3 like GRIM-19 and SOCS-1 are downregulated during cervical carcinogenesis [57], [58]. It is also important to note that apart from tumor cells' STAT3, a paracrine STAT3 activation in immune cells microenvironment may derive local MMP-9 expression which compliments to the pathobiology of human cervical high grade lesions [59] and somatic loss of STAT3 impairs HPV-induced tumorigenesis in cutaneous HPV models [60]. These aspects are being explored further in a separate investigation. Considering a central role of STAT3 in epithelial cell malignancies as well as above evidence, STAT3 apart from inducing expression of viral oncogenes E6 and E7, it may also mediate other HPV-independent carcinogenic events during cervical carcinogenesis.

Further, we investigated growth inhibitory events following inhibition of STAT3 phosphorylation by curcumin and AG490 that were associated with abrogated E6, E7 expression in HPV16 positive cervical cancer cells. Analysis of curcumin and AG490-treated cells by staining with Annexin V-propidium iodide, which can specifically distinguish live cells from cells undergoing apoptosis, revealed induction of apoptosis in cells with inhibited STAT3, which was further confirmed by results demonstrating cleavage of PARP1 and activation of central executer caspase-3 and loss of mitochondrial membrane potential. Active STAT3 is also responsible for a number of genes that promote cell proliferation and or prevent apoptotic cell death such as CyclinD1, myc, Bcl-xl, survivin, VEGF, mcl-1 [61]. Interestingly, expression of some of these genes such as Bcl-xl, survivin and Mcl-1 have been shown a strong correlation with pSTAT3 levels in cervical lesion [21] and their expression was found abrogated by specific targeting of STAT3 in cervical cancer cells [21], [22]. Therefore our results show that inhibition of STAT3 and loss of E6/E7 culminates in apoptotic cell death in HPV16 positive cervical cancer cells.

**Figure 6:**
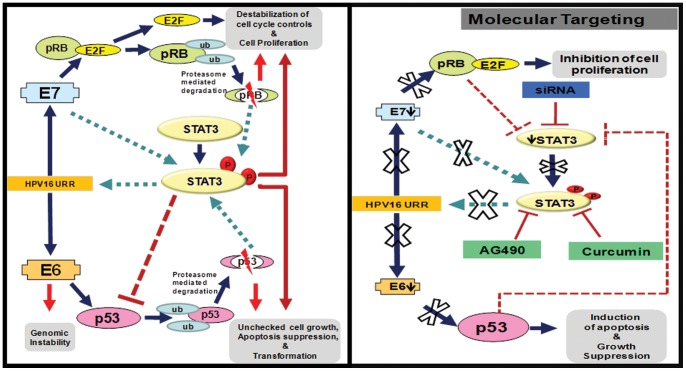
Schematic presentation of possible mechanism of STAT3 mediated transcriptional control of HPV16 E6 and E7 oncogene expression and effect of STAT3 targeting. Activated STAT3 binds to the HPV16 LCR and control the aberrant expression of E6 and E7 which further binds to cellular p53 and pRb and leads to their degradation during natural history of HPV16 infection (*left panel*). Control of STAT3 expression by specific siRNAs or STAT3 activation by specific pharmacological agents leads to the inhibition of STAT3 binding to the HPV16 LCR which results into suppression of E6 and E7 expression. Loss of E6 and E7 causes p53 and pRb accumulation and inhibition of cell proliferation with induction of apoptosis in cervical cancer cells (*right panel*). *Key; Solid thick arrows indicates positive upregulation; Solid thin arrows indicates loss of expression; dashed arrows indicates inhibition. small circles and boxes inidcates decreased level; big circles and boxes indicates upregulation.*

Overall, results obtained from the present investigation strongly support an important functional role of STAT3 in HPV16-mediated cervical carcinogenesis and inhibition of aberrantly overexpressed and constitutively activated STAT3 in early stage(s) of cervical cancer might prevent persistence of HPV16 infection, oncogenic transformation and progression of cervical lesions. Since the aberrant STAT3 activity observed in HPV16-induced cervical cancers is primarily manifested through persistent upstream signaling, in the absence of which STAT3 activity may revert back to its basal level, it gives a window of opportunity for effective therapeutic targeting by conventional herbal or rationally-designed STAT3 inhibitors (**Fig. 6**).
